# Safety, Tolerability, Pharmacokinetics, and Pharmacodynamics of the Neonatal Fc Receptor Inhibitor Rozanolixizumab: An Ethnic Sensitivity Study in Healthy Japanese, Chinese, and White Participants

**DOI:** 10.1002/cpdd.1484

**Published:** 2024-11-21

**Authors:** Assem el Baghdady, Rocío Lledó‐García, Maryam Gayfieva, Romana Lowcock, Shikiko Watanabe, Jagdev Sidhu, Denisa Wilkes

**Affiliations:** ^1^ UCB Slough UK; ^2^ Centre for Pharmaceutical Medicine Research Institute of Pharmaceutical Science King's College London London UK; ^3^ UCB Braine l'Alleud Belgium; ^4^ UCB Melbourne Australia; ^5^ Hammersmith Medicines Research London UK

**Keywords:** bioequivalence, ethnicity, FcRn, pharmacokinetic, rozanolixizumab

## Abstract

Rozanolixizumab is an anti‐human neonatal Fc receptor humanized immunoglobulin (Ig) G4 monoclonal antibody that reduces IgG, including pathogenic IgG autoantibodies. Rozanolixizumab safety and tolerability have been assessed in previous clinical studies with predominantly White participants. We assessed safety, tolerability, pharmacokinetics, and pharmacodynamics of single doses of rozanolixizumab in healthy Japanese and Chinese participants compared with White participants. This double‐blind, single‐center, UK‐based, Phase 1 study randomized 65 participants to rozanolixizumab 4 mg/kg (Japanese and White participants only), 7  mg/kg, or 10 mg/kg. All treatment‐emergent adverse events (TEAEs) were mild to moderate in severity; no severe TEAEs, serious TEAEs, or TEAEs leading to discontinuation occurred. Incidences of TEAEs in Japanese and Chinese participants were comparable to those in White participants. Japanese and Chinese participants had lower systemic rozanolixizumab exposure relative to Caucasian participants, attributable to lower actual doses administered due to lower body weight in Chinese and Japanese participants, indicating that body weight is not a relevant predictor of rozanolixizumab pharmacokinetics. All 3 ethnicities demonstrated dose‐dependent IgG reductions, with IgG nadir achieved around Day 10 and gradual return to baseline levels by Day 56. These data support the applicability of safety data from previous clinical studies of rozanolixizumab to individuals of Japanese and Chinese ethnicity.

Conducting Phase 1 studies across ethnic populations in healthy participants within a controlled environment enables identification of differences in pharmacokinetics (PK), safety, or tolerability of a drug. If ethnic differences are observed, dose adjustment can be considered before studies in larger patient populations are initiated. In addition, inclusive enrollment in clinical studies can also increase patients’ confidence in effective new treatments.^1^

Factors affecting small‐molecule drug disposition that can be influenced by ethnicity and cause differences in PK between ethnic populations include polymorphic enzymes involved in drug metabolism.^2^, ^3^ For monoclonal antibodies (mAbs) with linear PK characteristics, similar PK characteristics have been observed across ethnic groups.^4^ Target‐mediated drug disposition (TMDD) with nonlinear PK characteristics has been seen in some mAbs where the level of target binding impacts the disposition of the mAb^5^; in general, however, such differences for mAbs with TMDD across ethnicities are not observed.^6^, ^7^

Rozanolixizumab is a 148‐kDa humanized immunoglobulin (Ig) G4 monoclonal antibody that targets the neonatal Fc receptor (FcRn).^8^ Bound rozanolixizumab disrupts the interaction between IgG and FcRn, driving elimination of IgG, including pathogenic IgG autoantibodies, via the lysosomal degradation pathway, thus reducing IgG concentrations.^8^ IgG is therefore the pharmacodynamic (PD) parameter of interest for rozanolixizumab. Rozanolixizumab binds FcRn remotely from the albumin‐binding site,^8^ and no clinically meaningful decreases in mean albumin concentrations have been observed, indicating that albumin salvage remains intact.^9^

Rozanolixizumab demonstrated clinical benefit in the Phase 2 (MG0002; NCT03052751)^10^ and Phase 3 (MycarinG; NCT03971422)^9^ studies in patients with generalized myasthenia gravis. Rozanolixizumab is administered to patients via subcutaneous infusion^9^ and has previously been shown to elicit a nonlinear PK profile in human Phase 1 studies, suggestive of TMDD.^11^, ^12^ The Phase 1 and 2 studies were conducted in predominantly White populations.^10^, ^11^, ^13^ To support inclusion of a broader patient population in Phase 3 studies, we conducted a study to investigate the safety and tolerability of rozanolixizumab at varying dose levels and to characterize its PK and PD across healthy Japanese, Chinese, and White participants.

## Methods

### Study Design and Treatment Groups

This single‐center, Phase 1 clinical study (NCT03859219) was a randomized, double‐blind, placebo‐controlled, single‐ascending‐dose cohort study to assess the safety, tolerability, and PK of rozanolixizumab, and to explore the PD of a single dose of rozanolixizumab. Rozanolixizumab was administered by subcutaneous infusion (using a Cleo 90 infusion set [Smiths Medical] with Alaris^®^ GH Plus syringe pump [BD]) in healthy Japanese, Chinese, and White participants. The study took place between March 2019 and April 2020 at Hammersmith Medicines Research, London, UK.

**Figure 1:**
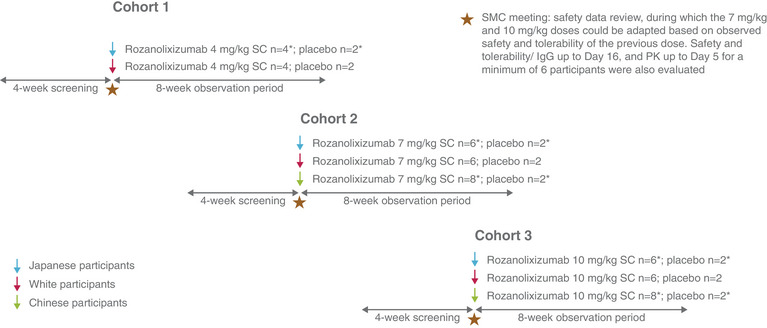
Study design incorporating 3 study cohorts, with a 4‐week screening prior to dosing with rozanolixizumab or placebo, followed by an 8‐week observation period. ^*^Following safety confirmation by the Safety Monitoring Committee of 2 sentinel participants per cohort (1 rozanolixizumab, 1 placebo; 48‐hour observation period), the remaining participants were randomized to receive rozanolixizumab or placebo. Placebo was a 0.9% sodium chloride aqueous solution, preservative free. Total number of study participants was 65. In the 4‐mg/kg cohort, there were 5 White participants instead of 4; an additional participant was recruited as 1 participant was withdrawn due to discontinuation of dosing after receiving 4% of the rozanolixizumab dose due to a technical issue. IgG, immunoglobulin G; PK, pharmacokinetics; SC, subcutaneous; SMC, Safety Monitoring Committee.

Participant eligibility was determined during a 4‐week screening period (Day −28 to Day −2) (Figure 1). Inclusion criteria were based on the familial heritage of study participants, where a Japanese, Chinese, or White participant must have been of relevant ethnic descent, based on the ethnicity of all 4 grandparents. Familial heritage for all ethnicities was confirmed verbally.

Eligible participants were admitted to the clinical site on Day −1 or the evening of Day −2 and entered a 6‐day in‐clinic period (Day −1 to Day 5). On Day 1, study participants were randomized to receive a single dose of subcutaneous placebo or rozanolixizumab in one of 3 cohorts of different rozanolixizumab doses. The 4‐mg/kg cohort included Japanese and White participants; the 7‐ and 10‐mg/kg cohorts included Japanese, White, and Chinese participants. Chinese participants were added to the study at the 7‐ and 10‐mg/kg dosing stage to align with Chinese regulatory guidance that required their inclusion at doses to be studied in Phase 3.

Study participants remained at the clinical site until Day 5; from Day 6 until the end of the study (Day 56), safety was assessed with participant visits to the clinical site on Days 7, 10, 13, 16, 19, 22, 29, 43, and 56. Safety oversight was provided by the Safety Monitoring Committee, who assessed 2 sentinel participants per cohort (1 rozanolixizumab, 1 placebo; 48‐hour observation period) for the Japanese and the Chinese cohorts. The remaining participants were randomized into one of 2 treatment groups per cohort, to receive rozanolixizumab or placebo and be dosed sequentially. No sentinel dosing for White participants was performed since sufficient safety data at higher doses were available from prior rozanolixizumab studies.^10^, ^11^, ^13^ The Safety Monitoring Committee confirmed each dose escalation prior to its initiation.

### Study Participants

All study participants who received rozanolixizumab (excluding a single withdrawn White participant) were included in the safety set; analysis of this set was according to treatment received by the participant. All study participants who received rozanolixizumab (excluding the single withdrawn White participant) were included in the PK per‐protocol set consisting of participants who did not have an important protocol deviation affecting PK parameters. All study participants (excluding the single withdrawn White participant) in the randomized set were included in the PD per‐protocol set consisting of participants who did not have an important protocol deviation affecting PD variables. Placebo study participants were pooled across all dose levels for safety and PD analysis.

### Study End Points

#### Safety

Safety end points were incidence of treatment‐emergent adverse events (TEAEs), vital sign measurements (blood pressure, pulse rate, respiratory rate, and temperature), hematology and biochemistry parameters, and 12‐lead electrocardiogram.

#### Pharmacokinetics, PD, and Immunology

PK parameters were based on plasma concentration of rozanolixizumab. IgG end points included the baseline‐corrected area under the total IgG response curve (AUC [IgG]), maximum percentage change from baseline in total IgG concentration and time to nadir (ie, occurrence of maximum percentage reduction in total IgG); and the observed values and change from baseline through the safety follow‐up period (to Day 56) in both total IgG and IgG subclasses. Other end points included observed values and change from baseline through the safety follow‐up period (to Day 56) in serum Ig concentrations of total IgA, IgE, and IgM, albumin concentration, and anti‐drug antibody (ADA) screening status (positive or negative) at each scheduled assessment during the in‐clinic and safety follow‐up periods.

### Study Ethics

This study was conducted in compliance with the current version of the applicable regulatory and International Council for Harmonization Good Clinical Practice requirements and with the principles expressed in the Declaration of Helsinki (version dated October 2013). The final research protocol and the informed consent were approved by the UK Medicines and Healthcare Products Regulatory Agency and the locally appointed ethics committee (Health and Social Care Research Ethics, Office for Research Ethics Committee Northern Ireland, UK), and written consent was obtained from all participants prior to the screening activities of the study.

### Study Assessments and Statistics

#### Sample Analysis Methods for PK Data

Blood samples for PK analysis were taken before dosing; immediately after the end of infusion; at 4, 6, 8, 12, 24, 36, 48, 72, and 96 hours after dosing; and on Days 7 and 10. Samples were stored at −80°C. When access to study sites for site monitoring was limited due to COVID‐19, remote monitoring was applied in line with guidance from the UK Medicines and Healthcare Products Regulatory Agency. The concentration of rozanolixizumab in lithium heparin plasma was determined by electrochemiluminescence immunoassay based on a double‐antibody sandwich method, MSD (see Supplemental Methods for full methods). The validated range of the assay was 200‐12,800 ng/mL. All data collection was carried out using MSD Discovery Workbench software version 4.0.12.1 (MSD). Raw data were imported into Watson software version 7.2 (ThermoFisher Scientific) for regression quantification and storage. Noncompartmental analysis was undertaken using Phoenix WinNonlin software version 8.1 (Certara), to calculate area under the curve from time 0 to the last measurable concentration (AUC_0‐t_), maximum concentration (C_max_), time to maximum concentration, and the same parameters normalized by dose amount and/or body weight.

#### Sample Analysis Methods for PD Data

Blood samples for PD analysis were taken at screening; before dosing; at 24, 48, 72, and 96 hours after dosing; and on Days 7, 10, 13, 16, 19, 22, 29, 43, and 56. Concentration of IgG was determined by immunoturbidimetric assay using a Tina‐quant Immunoglobulin G Gen.2 commercial kit (Roche), according to the manufacturer's instructions (see Supplemental Methods). The measuring range of the assay was 3.00‐50.0 g/L IgG.

For detection of IgG Subclasses 1‐4, a commercially available Optilite kit (Binding Site Group Ltd.) was used according to the manufacturer's instructions. Readouts were measured using a Binding Site Optilite Analyser (Binding Site Group Ltd.).

For quantification of albumin, serum samples were analyzed by standard techniques using a COBAS C702 clinical analyzer (Roche).

#### Sample Analysis Methods for Detection of ADAs

Immunogenicity testing occurred via a tiered analysis approach consisting of a screening, confirmatory, and titration assay (see Supplemental Methods). Following a risk‐based approach, the screening tier of an ADA assay was designed statistically to yield an approximately 5% false‐positive rate. Any sample with a signal greater than 1.08 × mean plate negative control was considered positive in the screening assay and tested in the confirmatory tier of the assay.

The concentration of ADA in human lithium heparin plasma was determined by electrochemiluminescent immunoassay. All data collection was carried out using MSD Discovery Workbench software version 4.0.12.1. Data storage was performed using the software package Watson version 7.2 (ThermoFisher Scientific).

### Statistical Analyses

Safety, tolerability, PK, PD, and ADA data were summarized using descriptive statistics and graphical analyses by ethnicity within each cohort. Data from Japanese and Chinese participants were statistically compared with White participants, but no formal statistical comparisons between Japanese and Chinese participants were undertaken.

The primary comparison of interest was to estimate the similarity of plasma PK parameters (C_max_ and AUC_0‐t_) between Japanese and White, and Chinese and White groups by dose levels, using an analysis of variance (ANOVA) model with ethnicity as a fixed effect fitted to the log‐transformed PK parameters. Body‐weight‐normalized AUC_0‐t_ and C_max_ were compared between Japanese and White, and Chinese and White groups using an ANOVA on the log‐transformed parameters. A similar approach was used for comparing dose‐normalized AUC_0‐t_ and C_max_. Geometric mean profiles of plasma concentrations with 95% confidence interval (CI) are presented by ethnic group for each dose level. Point estimates for the ratio of geometric means between Japanese or Chinese (test) and White (reference) study participants and their respective 2‐sided 90% CIs were computed using the least square means and the root mean squares of error from the analysis of covariance (ANCOVA).

The comparison of IgG baseline‐corrected AUC between Japanese and White, and Chinese and White groups was assessed using an ANCOVA model with ethnicity as a fixed effect and baseline value as a covariate fitted to the baseline‐corrected AUC (IgG). Point estimates for the difference in least square means between Japanese or Chinese (test) and White (reference) study participants and their respective 2‐sided 95% CIs were computed using the least square means and the root mean squares of error from the ANCOVA. Percentage change in total IgG or IgG subclasses from baseline with 95% CI are presented by ethnic group for each dose level.

## Results

### Participant Demographics

Of the 134 participants screened, 65 were randomized and participated in the study (Japanese, n = 22; Chinese, n = 20; White, n = 23) (Table 1); 64 participants completed the study. One White participant in the rozanolixizumab 4‐mg/kg dose group was withdrawn from the study due to a technical issue that resulted in the participant receiving approximately 4% of the planned dose. For this reason, a fifth White participant was included in this cohort such that 4 patients receiving rozanolixizumab 4 mg/kg completed the study. This study ended in April 2020. No Japanese or White participants had protocol deviations related to the COVID‐19 pandemic; 4 Chinese participants had COVID‐19‐related protocol deviations, none of which led to exclusion from either the PK or PD analysis.

**Table 1:** Baseline Demographics for Japanese, Chinese, and White Participants, Including Predose Values for Total IgG

	Rozanolixizumab 4 mg/kg		Rozanolixizumab 7 mg/kg			Rozanolixizumab 10 mg/kg			Placebo			All		
	Japanese, n = 4	White, n = 5	Japanese, n = 6	Chinese, n = 8	White, n = 6	Japanese, n = 6	Chinese, n = n = 8	White, n = 6	Japanese, n = 6	Chinese, n = 4	White, n = 6	Japanese, n = 22	Chinese, n = 20	White, n = 23
Age (years), median (range)	31.0 (29‐42)	45.0 (24‐60)	29.0 (23‐54)	33.0 (22‐49)	41.0 (32‐54)	30.0 (28‐49)	36.0 (20‐46)	33.5 (20‐55)	29.5 (22‐59)	30.5 (21‐47)	45.5 (35‐54)	29.0 (22‐59)	35.0 (20‐49)	42.0 (20‐60)
Female, n (%)	4 (100)	0	6 (100)	4 (50)	3 (50)	2 (33)	4 (50)	3 (50)	5 (83)	1 (25)	3 (50)	17 (77)	9 (45)	9 (39)
Male, n (%)	0	5 (100)	0	4 (50)	3 (50)	4 (67)	4 (50)	3 (50)	1 (17)	3 (75)	3 (50)	5 (23)	11 (55)	14 (61)
Weight (kg), mean (SD)	54.8 (3.9)	78.1 (12.4)	53.3 (5.1)	62.6 (12.5)	73.5 (11.9)	62.1 (12.0)	63.7 (8.8)	78.9 (7.9)	57.0 (9.2)	71.2 (19.3)	78.5 (5.3)	57.0 (8.7)	64.7 (12.5)	77.2 (9.3)
Height (cm), mean (SD)	160.3 (5.3)	177.2 (4.4)	160.7 (3.4)	169.5 (10.2)	171.3 (9.9)	165.5 (8.7)	164.0 (5.9)	175.3 (6.8)	162.2 (9.0)	172.5 (6.4)	170.3 (9.7)	162.3 (6.9)	167.9 (8.4)	173.4 (8.1)
BMI (kg/m^2^), mean (SD)	21.4 (1.9)	24.8 (2.9)	20.6 (1.5)	21.6 (2.5)	24.9 (1.8)	22.5 (3.0)	23.6 (1.9)	25.6 (1.8)	21.6 (2.6)	23.7 (5.5)	27.2 (2.7)	21.6 (2.3)	22.8 (3.1)	25.7 (2.4)
Geometric mean total IgG (95% CI) (g/L)	10.86 (8.2, 14.4)	8.98 (6.2, 12.9)	11.62 (10.9, 12.4)	12.61 (12.0, 13.3)	9.46 (7.5, 12.0)	10.49 (9.0, 12.2)	11.62 (10.2, 13.2)	9.19 (7.3, 11.5)	11.43 (10.6, 12.4)	10.90 (9.3, 12.8)	10.78 (9.5, 12.2)	NR	NR	NR
Total IgG (g/L), mean (SD)	11.0 (1.8)	9.1 (2.0)	11.6 (0.7)	12.6 (0.7)	9.7 (2.2)	10.6 (1.5)	11.7 (1.7)	9.4 (2.0)	11.5 (0.8)	10.9 (1.1)	10.8 (1.3)	NR	NR	NR

BMI, body mass index; CI, confidence interval; Ig, immunoglobulin; NR, not reported; SD, standard deviation.

Japanese participants were predominantly women (100% in the 4‐ and 7‐mg/kg dose groups; 77% overall); 50% of Chinese and White participants were women for 7‐ and 10‐mg/kg dose cohorts. The median ages of Japanese, Chinese, and White participants were 29.0, 35.0, and 42.0 years, respectively.

Japanese and Chinese participants had a lower median body weight (54.8 and 63.9 kg, respectively) compared with White participants (77.7 kg), resulting in Japanese and Chinese participants receiving generally lower absolute doses of rozanolixizumab than White participants; the total dose a participant received was calculated on the basis of their body weight.

### Safety

Overall, single subcutaneous doses of 7‐ and 10‐mg/kg rozanolixizumab were tolerated with an acceptable safety profile across all ethnicities; a single subcutaneous dose of 4 mg/kg rozanolixizumab was also assessed in Japanese and White participants and found to be tolerated with an acceptable safety profile. There were no serious, severe, or fatal TEAEs, and none of the participants discontinued the study due to TEAEs (Table S1).

The observed TEAEs were consistent with the known rozanolixizumab safety profile.^9^, ^10^, ^11^, ^13^ Across all study groups, a numerically higher incidence of TEAEs was reported for those receiving rozanolixizumab (38 participants [78%] reported 115 TEAEs) compared with placebo (10 participants [63%] reported 23 TEAEs). Japanese and Chinese participants receiving rozanolixizumab reported a similar incidence of TEAEs (75% and 81%, respectively) as the White participants (77%). In placebo groups, the incidence of TEAEs was 67% (4/6) for Japanese participants, 50% (2/4) for Chinese participants, and 67% (4/6) for White participants.

The most frequently reported TEAEs across all ethnic groups were headache, infusion‐site erythema, nasopharyngitis, and dizziness (Table S1). All TEAEs reported by study participants were either of mild or moderate intensity. In White participants, the highest incidence of headache was observed in the rozanolixizumab 10‐mg/kg dose group (67%). In Japanese and Chinese participants, incidence of headache in the 10‐mg/kg dose group (17% and 38%, respectively) was comparable to that with placebo (17% and 50%, respectively). The majority of events of headache had an onset 1‐3 days after infusion, were resolved within 1 day, and were managed with paracetamol and/or ibuprofen. Overall, 37% (18/49) and 6.3% (1/16) of participants in the rozanolixizumab and placebo treatment groups reported infusion‐site reactions, respectively. The most common infusion‐site reaction reported among participants in the rozanolixizumab group was infusion‐site erythema (29% [14/49). All injection‐site reactions were of mild severity and resolved without sequelae.

Infections were reported by 2 Japanese study participants (13%), 4 Chinese study participants (25%), and 3 White participants (18%) receiving rozanolixizumab. All reported infections were mild or moderate in intensity. The most common infection was nasopharyngitis.

Mean vital sign measurements (blood pressure [systolic and diastolic], pulse rate, respiratory rate, and temperature), hematology and biochemistry parameters, and 12‐lead electrocardiogram results remained within the normal ranges over time in all ethnic groups.

### Pharmacokinetics

**Figure 2:**
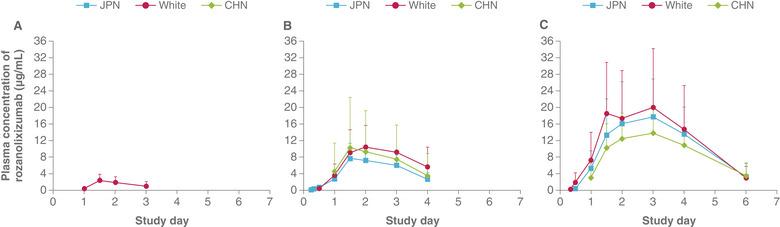
Plasma rozanolixizumab concentrations in Japanese, Chinese, and White participants, following subcutaneous administration of rozanolixizumab 4 mg/kg (A), 7 mg/kg (B) or 10 mg/kg (C). Data reported as arithmetic mean; error bars represent standard deviation. CHN, Chinese; JPN, Japanese.

All study participants who received rozanolixizumab, except for 1 White participant from Cohort 1 who received only 4% of the planned dose, were included in the PK analyses (n = 48). Plasma rozanolixizumab concentrations were quantifiable in 2 of 4 Japanese participants on 4 mg/kg at Day 2, and in all 4 White participants for this same dose at Day 1 (Figures 2 and S1, Table 2). Mean systemic rozanolixizumab exposure was lower in both Japanese and Chinese participants relative to White participants at 7 mg/kg and, to a lesser extent, at 10 mg/kg. Nonlinearity was observed when comparing derived PK parameters such as C_max_/dose across the dosing groups within each ethnic group, where the parameter was supra‐proportional with increasing dose (Table 2). The large interindividual variability of PK across all ethnic groups resulted in a large interindividual coefficient of variation, pooled across ethnicities, in the ANOVA results (Table S2). Compared with White participants, there was a trend to lower rozanolixizumab C_max_ and AUC_0‐t_ in Japanese and Chinese participants at all dose levels; when adjusted for actual administered dose, the differences in PK parameters were reduced. Overall, based on the ANOVA, no differences can be concluded across ethnicities.

**Table 2:** PK End Points for Plasma Rozanolixizumab in Japanese, Chinese, and White Participants, Following Subcutaneous Administration of 4, 7, or 10 mg/kg Rozanolixizumab (PK Per‐Protocol Set)

	Rozanolixizumab 4 mg/kg		Rozanolixizumab 7 mg/kg			Rozanolixizumab 10 mg/kg		
	Japanese, n = 4	White, n = 4	Japanese, n = 6	Chinese, n = 8	White, n = 6	Japanese, n = 6	Chinese, n = 8	White, n = 6
C_max_ (µg/mL)								
Geometric mean (95% CI)	–^a^	2.43 (0.96‐6.12)	7.08 (3.37‐14.88)	5.16 (1.49‐17.95)	9.41 (4.39‐20.13)	13.88 (4.53‐42.51)	13.09 (8.34‐20.53)	19.74 (10.39‐37.49)
Arithmetic mean (SD)	0.34 (0.49)	2.71 (1.31)	8.16 (3.19)	10.71 (12.02)	11.13 (5.49)	18.34 (9.05)	14.50 (5.83)	22.87 (12.66)
t_max_ (day), median (range)	–^a^	1.5 (1.5‐2.0)	1.5 (1.5‐3.0)	2.0 (1.5‐4.0)	2.0 (1.5‐3.0)	3.0 (1.5‐4.0)	3.0 (2.0‐4.0)	3.0 (1.5‐3.0)
AUC_(0‐t)_ (day·µg/mL)								
Geometric mean (95% CI)	–^a^	2.89 (0.66‐12.75)	16.79 (9.33‐30.22)	10.80 (2.95‐39.52)	21.62 (7.86‐59.46)	44.78 (14.05‐142.7)	39.64 (21.06‐74.60)	52.37 (21.55‐127.2)
Arithmetic mean (SD)	0.26 (0.39)	3.79 (2.77)	18.53 (6.81)	23.90 (28.37)	28.81 (18.18)	60.10 (30.34)	46.97 (21.03)	68.20 (47.48)
BW‐normalized C_max_ (µg/mL/kg)								
Geometric mean (95% CI)	–^a^	0.0313 (0.01‐0.08)	0.132 (0.07‐0.27)	0.0840 (0.03‐0.28)	0.129 (0.06‐0.26)	0.227 (0.08‐0.66)	0.207 (0.13‐0.34)	0.251 (0.13‐0.49)
Arithmetic mean (SD)	0.00663 (0.00983)	0.0345 (0.0143)	0.151 (0.0580)	0.162 (0.169)	0.150 (0.0750)	0.294 (0.146)	0.235 (0.110)	0.294 (0.166)
BW‐normalized AUC_(0‐t)_ (day•µg/mL/mg)								
Geometric mean (95% CI)	–^a^	0.0373 (0.01‐0.15)	0.316 (0.18‐0.55)	0.176 (0.05‐0.61)	0.297 (0.11‐0.78)	0.732 (0.24‐2.20)	0.628 (0.33‐1.21)	0.667 (0.28‐1.60)
Arithmetic mean (SD)	0.00508 (0.00787)	0.0464 (0.0278)	0.345 (0.127)	0.361 (0.397)	0.394 (0.279)	0.962 (0.482)	0.765 (0.404)	0.871 (0.631)
Dose‐normalized C_max_ (µg/mL/mg)								
Geometric mean (95% CI)	–^a^	0.00785 (0.00‐0.02)	0.0191 (0.01‐0.04)	0.0122 (0.004‐0.04)	0.0185 (0.01‐0.04)	0.0227 (0.01‐0.07)	0.0207 (0.01‐0.03)	0.0251 (0.01‐0.05)
Arithmetic mean (SD)	0.00166 (0.00247)	0.00863 (0.00359)	0.0216 (0.00829)	0.0243 (0.0269)	0.0214 (0.0107)	0.0295 (0.0146)	0.0235 (0.0110)	0.0294 (0.0166)
Dose‐normalized AUC_0‐t_ (day•µg/mL/mg)								
Geometric mean (95% CI)	–^a^	0.00934 (0.002‐0.04)	0.0452 (0.03‐0.08)	0.0255 (0.01‐0.09)	0.0425 (0.02‐0.11)	0.0733 (0.02‐0.22)	0.0628 (0.03‐0.12)	0.0667 (0.03‐0.16)
Arithmetic mean (SD)	0.00128 (0.00197)	0.0116 (0.00696)	0.0494 (0.0182)	0.0542 (0.0635)	0.0562 (0.0398)	0.0963 (0.0483)	0.0766 (0.0405)	0.0872 (0.0632)

AUC_0‐t_, area under the curve from time 0 to the last measurable concentration; BMI, body mass index; BW, body weight; CI, confidence interval; C_max_, maximum concentration; PK, pharmacokinetics; SD, standard deviation; t_max_, time to maximum concentration.

a Only 2 participants were included for this measurement; 2 participants were excluded since their individual value was recorded as 0. Therefore, the geometric mean and CI are not calculable for this group.

### Pharmacodynamics

**Figure 3:**
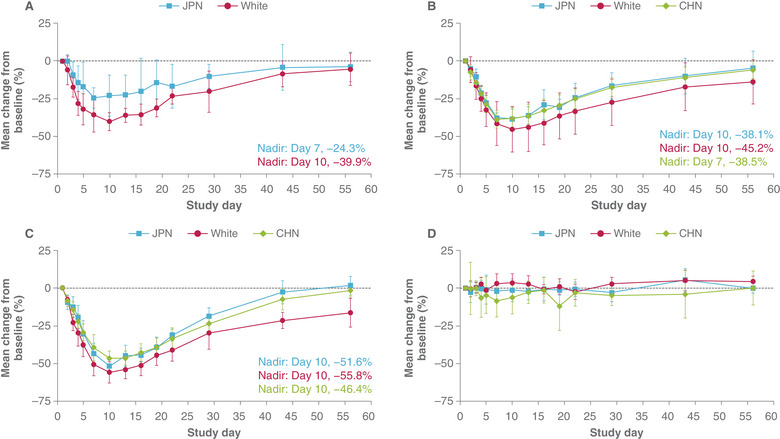
Mean percentage change in total IgG from baseline‐time profiles in Japanese, Chinese, and White participants, following subcutaneous administration of rozanolixizumab 4 mg/kg (A), 7 mg/kg (B) or 10 mg/kg (C), or placebo (D). Error bars represent 95% confidence interval. CHN, Chinese; JPN, Japanese.

All study participants (except for 1 White participant from Cohort 1) were included in PD analyses (n = 64). Following single doses of rozanolixizumab, dose‐dependent reductions in total blood IgG concentration were observed in all ethnicities (Figure 3), concordant with plasma rozanolixizumab concentration changes with doses. IgG nadir was reached around Day 10 with a gradual return to baseline levels by Day 56. Mean percentage reduction in total IgG (R_min_ value, independent of the day) was 47%, 52%, and 56% in the 10‐mg/kg dose cohort for Chinese, Japanese, and White participants, respectively, where R_min_ is the maximum decrease in total plasma IgG (absolute percentage change from baseline).

Similar profiles, albeit with lower magnitude of response, were observed for the 4‐mg/kg (assessed in Japanese and White participants) and 7‐mg/kg doses of rozanolixizumab. ANCOVA results demonstrated that baseline‐corrected reductions in AUC (IgG) were overall slightly greater in White participants relative to Japanese and Chinese participants (Table S3).

### Immunology

In the IgG subclasses, the largest dose‐dependent reduction was observed in subclass 3 (Figure S2), although for all doses, reductions in IgG subclasses generally mirrored those for total serum IgG concentration. In comparison, there were no clinically significant changes to IgA, IgE, or IgM following rozanolixizumab administration in any ethnic group (data not shown). The mean albumin value remained within the normal range over time in line with placebo, with no clinically relevant changes from baseline across all dose cohorts following rozanolixizumab administration (Figure S3).

No study participants were ADA positive at baseline. Treatment‐emergent ADA positivity was observed at Day 22 in 1 participant (Japanese, 7 mg/kg group), and no TEAEs associated with emergent ADAs were observed in this participant.

## Discussion

This study demonstrated the comparability of the safety, tolerability, PK, and PD of a single weight‐based dose of rozanolixizumab between 2 Asian ethnicity populations and a White population. TEAEs, plasma concentration of rozanolixizumab and blood IgG concentration in healthy Japanese and Chinese participants were comparable to those in White participants following subcutaneous infusion of rozanolixizumab. Rozanolixizumab was tolerated with an acceptable safety profile in Japanese, Chinese, and White study participants following single subcutaneous administration of 7 and 10 mg/kg doses, and of 4 mg/kg as assessed in Japanese and White participants. No serious, severe or fatal TEAEs were reported in any ethnic group during this study, and no participants discontinued the study due to TEAEs. Of note, mean albumin level remained within the normal range over time in line with placebo, with no clinically relevant changes from baseline observed across ethnic and dose groups. While earlier studies of rozanolixizumab included a small number of patients of non‐White ethnicity, no new safety concerns were identified, and no unexpected findings were observed, compared with the rozanolixizumab Phase 1,^11^ Phase 2,^10^, ^13^ or Phase 3^9^ studies.

Rozanolixizumab PK characteristics were consistent with those observed in previous studies^9^, ^10^, ^11^, ^13^ in demonstrating a rapid clearance of rozanolixizumab driven by its mechanism of action of blocking FcRn,^8^ its own recycling system. The PK data reflect the nonlinear TMDD characteristics of rozanolixizumab, which have also been reported with other mAb‐based drugs.^5^ The lower systemic rozanolixizumab exposure observed in both Japanese and Chinese participants, relative to White participants, is likely due to lower administered doses (mg) of rozanolixizumab administered in accordance with lower body weights in the Asian participants. Relative to White participants, point estimates for the ratio of geometric means of AUC_0‐t_ and C_max_ for both Japanese and Chinese participants across the dose groups were less than 1, although the 90% CIs for each parameter in the 7‐ and 10‐mg/kg dose groups contained the value of 1. When these parameters were adjusted for body weight or the dose administered, comparable exposure parameters, with ratios closer to 1, were observed overall for the 7‐ and 10‐mg/kg dose cohorts.

Overall systemic exposure in the Chinese 7‐mg/kg dose group was impacted by unexpectedly low exposures in 2 participants (C_max_ values of 0.99 and 0.44 µg/mL and AUC_0‐t_ values of 1.48 and 1.04 day•µg/mL, respectively). There were no study operational reasons for these low exposures. Further, the lack of quantifiable systemic rozanolixizumab concentrations in 2 of 4 participants in the 4‐mg/kg Japanese group prevented meaningful interpretation of PK data and comparisons in this cohort. Such unpredictable low or nonquantifiable exposures of unbound systemic rozanolixizumab can be encountered due to rozanolixizumab's TMDD characteristics. The PK data in this study, arising from milligram/kilogram dosing, demonstrate that milligram/kilogram dosing does not reduce variability in systemic rozanolixizumab exposures between individuals. A fixed milligram dose for individuals in prespecified body weight ranges as defined in the approved dosing for rozanolixizumab is expected to maintain consistency in systemic exposures.

Following subcutaneous infusion of rozanolixizumab, dose‐dependent reductions in IgG were observed for all ethnicities. IgG nadir was achieved around Day 10, and overall systemic exposure outcomes observed for each cohort aligned with the percentage change from baseline in IgG. Systemic exposure to rozanolixizumab was lower in Japanese and Chinese participants than in White participants in the 7‐ and 10‐mg/kg dose groups, and lower in Japanese than in White participants in the 4‐mg/kg dose group; however, baseline‐corrected AUCs for IgG were similar between ethnic groups. One Japanese participant in the rozanolixizumab 7‐mg/kg dose group was ADA positive on Day 22, with no concurrent TEAEs; the low occurrence of ADAs was in line with expectations based on the Phase 1 study.^11^

A limitation of this study is the relatively small subgroup sizes relative to the PK variability of rozanolixizumab; however, population sizes were sufficient to assess PD outcomes, in the form of IgG assessment. Ethnicity assessment in a healthy population restricts this study to a single dose of rozanolixizumab, with a highest dose of 10 mg/kg. In common with many studies during this period, precautions during the COVID‐19 pandemic resulted in a small degree of procedural noncompliance, such as the assessment visit taking place as a phone call; however, none of these were considered clinically significant.

In conclusion, subcutaneous infusions of rozanolixizumab doses up to 10 mg/kg were tolerated, with an acceptable safety profile in all ethnic populations. Rozanolixizumab showed dose‐dependent reductions in IgG within both Japanese and Chinese ethnic populations that were comparable to the White population, with similar baseline‐corrected AUCs for IgG between ethnic groups. These data support the applicability of safety data from clinical studies of rozanolixizumab with predominantly White populations to Japanese and Chinese patients and support the inclusion of participants in these regions in Phase 3 studies.^9^

## Conflicts of Interest

Assem el Baghdady is an external consultant contracted by UCB and Senior Lecturer, Centre for Pharmaceutical Medicine Research, King's College London. Rocío Lledó‐García, Maryam Gayfieva, Jagdev Sidhu, and Shikiko Watanabe are employees and stockholders of UCB. Romana Lowcock was a Veramed consultant for UCB during the conduct of the study. Veramed is a full‐service provider for UCB. Romana Lowcock is currently employed by ONO Pharma UK Ltd. Denisa Wilkes has no conflicts to declare.

## Funding

This research was funded by UCB. Medical writing support was provided by Jenny Fanstone and Karen Mansfield of Ogilvy Health, Oxford, UK, and funded by UCB, in accordance with Good Publications Practice guidelines (http://www.ismpp.org/gpp3).

## Supporting information



Supporting Information
