# Supplementary material for: Safety, Tolerability, Pharmacokinetics, and Pharmacodynamics of the Neonatal Fc Receptor Inhibitor Rozanolixizumab: An Ethnic Sensitivity Study in Healthy Japanese, Chinese, and White Participants
Source: Clin Pharmacol Drug Dev. 2024 Nov 21;14(1):26–35. doi: 10.1002/cpdd.1484 (PMC11701949; doi:10.1002/cpdd.1484)
Supplement: Supplementary file 1 — Supporting Information [file CPDD-14-26-s001.docx]

# Supplementary Tables

## Supplementary Table S1: Safety profile of rozanolixizumab in Japanese (JPN), Chinese (CHN) and White participants, following subcutaneous infusion of 4 mg/kg, 7 mg/kg or 10 mg/kg rozanolixizumab, or administration of placebo (Safety Set)

|  | **Rozanolixizumab**  **4 mg/kg** | | **Rozanolixizumab**  **7 mg/kg** | | | **Rozanolixizumab**  **10 mg/kg** | | | **Placebo**  **total** | | | **Rozanolixizumab**  **total** | | |
| --- | --- | --- | --- | --- | --- | --- | --- | --- | --- | --- | --- | --- | --- | --- |
| **n, (%)** | **JPN**  **n=4** | **White**  **n=5** | **JPN**  **n=6** | **CHN**  **n=8** | **White**  **n=6** | **JPN**  **n=6** | **CHN**  **n=8** | **White**  **n=6** | **JPN**  **n=6** | **CHN**  **n=4** | **White**  **n=6** | **JPN**  **N=16** | **CHN**  **N=16** | **White**  **N=17** |
| **Any TEAEs** | 2  (50) | 3  (60) | 5  (83) | 5  (63) | 5  (83) | 5  (83) | 8  (100) | 5  (83) | 4  (67) | 2  (50) | 4  (67) | 12  (75) | 13  (81) | 13  (77) |
| **Serious TEAEs** | 0 | 0 | 0 | 0 | 0 | 0 | 0 | 0 | 0 | 0 | 0 | 0 | 0 | 0 |
| **Treatment-related TEAEs** | 0 | 1  (20) | 5  (83) | 4  (50) | 5  (83) | 5  (83) | 7  (88) | 5  (83) | 3  (50) | 2  (50) | 2  (33) | 10  (63) | 11  (69) | 11  (65) |
| **Severe TEAEs** | 0 | 0 | 0 | 0 | 0 | 0 | 0 | 0 | 0 | 0 | 0 | 0 | 0 | 0 |
| **Deaths** | 0 | 0 | 0 | 0 | 0 | 0 | 0 | 0 | 0 | 0 | 0 | 0 | 0 | 0 |
| **Most common TEAEs (≥10% of patients receiving rozanolixizumab)** |  |  |  |  |  |  |  |  |  |  |  |  |  |  |
| **Headache** | 0 | 0 | 3  (50) | 2  (25) | 3  (50) | 1  (17) | 3  (38) | 4  (67) | 1  (17) | 2  (50) | 1  (17) | 4  (25) | 5  (31) | 7  (41) |
| **Infusion-site erythema** | 0 | 0 | 2  (33) | 1  (13) | 4  (67) | 2  (33) | 3  (38) | 2  (33) | 1  (17) | 0 | 0 | 4  (25) | 4  (25) | 6  (35) |
| **Nasopharyngitis** | 1  (25) | 0 | 0 | 2  (25) | 0 | 1  (17) | 2  (25) | 0 | 0 | 1  (25) | 1  (17) | 2  (13) | 4  (25) | 0 |
| **Dizziness** | 1  (25) | 0 | 2  (33) | 1  (13) | 0 | 1  (17) | 0 | 0 | 1  (17) | 0 | 0 | 4  (25) | 1  (6) | 0 |

TEAE, treatment-emergent adverse event.

## Supplementary Table S2: Summary of ANOVA for PK parameters of rozanolixizumab in Japanese (JPN), Chinese (CHN) and White participants (PK per-protocol set)

|  | **Rozanolixizumab**  **7 mg/kg** | | **Rozanolixizumab**  **10 mg/kg** | |
| --- | --- | --- | --- | --- |
|  | **Ratio JPN/White** | **Ratio CHN/White** | **Ratio JPN/White** | **Ratio CHN/White** |
| **C_max_ μg/mL** |  |  |  |  |
| **Estimate** | 0.75 | 0.55 | 0.70 | 0.66 |
| **90% CI** | 0.25, 2.28 | 0.19, 1.55 | 0.33, 1.50 | 0.33, 1.34 |
| **ANOVA CV*, %** | 154.1 | | 87.0 | |
| **AUC_(0–t)_, d**·**μg/mL** |  |  |  |  |
| **Estimate** | 0.78 | 0.50 | 0.86 | 0.76 |
| **90% CI** | 0.24, 2.50 | 0.17, 1.49 | 0.35, 2.11 | 0.33, 1.76 |
| **ANOVA CV*, %** | 169.9 | | 111.2 | |
| **BW-normalized C_max_ μg/mL/kg** |  |  |  |  |
| **Estimate** | 1.03 | 0.65 | 0.90 | 0.82 |
| **90% CI** | 0.36, 2.96 | 0.24, 1.75 | 0.43, 1.90 | 0.41, 1.66 |
| **ANOVA CV*, %** | 142.3 | | 86.3 | |
| **BW-normalized AUC_(0–t)_, d**·**μg/mL/kg** |  |  |  |  |
| **Estimate** | 1.06 | 0.59 | 1.10 | 0.94 |
| **90% CI** | 0.35, 3.26 | 0.21, 1.69 | 0.45, 2.67 | 0.41, 2.16 |
| **ANOVA CV*, %** | 157.2 | | 109.0 | |
| **Dose-normalized C_max_ μg/mL/mg** |  |  |  |  |
| **Estimate** | 1.03 | 0.66 | 0.90 | 0.83 |
| **90% CI** | 0.35, 3.00 | 0.24, 1.79 | 0.43, 1.91 | 0.41, 1.66 |
| **ANOVA CV*, %** | 144.9 | | 86.2 | |
| **Dose-normalized AUC_(0–t)_, d**·**μg/mL/mg** |  |  |  |  |
| **Estimate** | 1.06 | 0.60 | 1.10 | 0.94 |
| **90% CI** | 0.34, 3.30 | 0.21, 1.73 | 0.45, 2.67 | 0.41, 2.16 |
| **ANOVA CV*, %** | 160.2 | | 109.0 | |

*Inter-individual CV, pooled across ethnicities.

ANOVA, analysis of variance; AUC_(0–t)_, area under the curve up to the last measurable concentration; BW, body weight; CI, confidence interval; CV, coefficient of variation; PK, pharmacokinetic.

## Supplementary Table S3: Summary of ANCOVA for IgG baseline-corrected AUC in White, Japanese (JPN) and Chinese (CHN) participants, following subcutaneous administration of 4 mg/kg, 7 mg/kg or 10 mg/kg rozanolixizumab

| **Baseline corrected (g*day/L)** | **JPN**  **LS mean**  **(SE)** | **White**  **LS mean**  **(SE)** | **JPN vs White**  **estimate**  **(95% CI)** | **CHN**  **LS mean**  **(SE)** | **White**  **LS mean**  **(SE)** | **CHN vs White**  **estimate**  **(95% CI)** |
| --- | --- | --- | --- | --- | --- | --- |
| **Placebo** | 2.144  (8.706) | 14.89  (8.599) | −12.75  (−39.85, 14.36) | −22.51  (10.44) | 14.89  (8.599) | −37.40  (−66.68, −8.118) |
| **Rozanolixizumab**  **4 mg/kg** | −62.52  (15.47) | −97.46  (15.47) | 34.94  (−24.89, 94.77) | – | – | – |
| **Rozanolixizumab**  **7 mg/kg** | −124.0  (21.41) | −123.2  (27.21) | −0.805  (−76.15, 74.54) | −142.7  (21.63) | −123.2  (27.21) | −19.47  (−104.0, 65.03) |
| **Rozanolixizumab**  **10 mg/kg** | −120.4  (10.94) | −177.9  (11.83) | 57.46  (22.94, 91.98) | −136.0  (10.76) | −177.9  (11.83) | 41.87  (5.67, 78.08) |
| **Rozanolixizumab total** | −106.4  (11.21) | -140.7  (12.70) | 34.29  (−0.63, 69.21) | −136.8  (12.70) | −140.7  (12.70) | 3.96  (−35.70, 43.62) |

ANCOVA, analysis of covariance; AUC, area under the curve; CI, confidence interval; Ig, immunoglobulin; LS, least-squares; PD, pharmacodynamics; SE, standard error.

# Supplementary Figures

## Supplementary Figure S1: Total amount of plasma rozanolixizumab in mg: distribution per ethnic group, following subcutaneous (SC) administration of single doses (SD) of rozanolixizumab 4 mg/kg, 7 mg/kg or 10 mg/kg


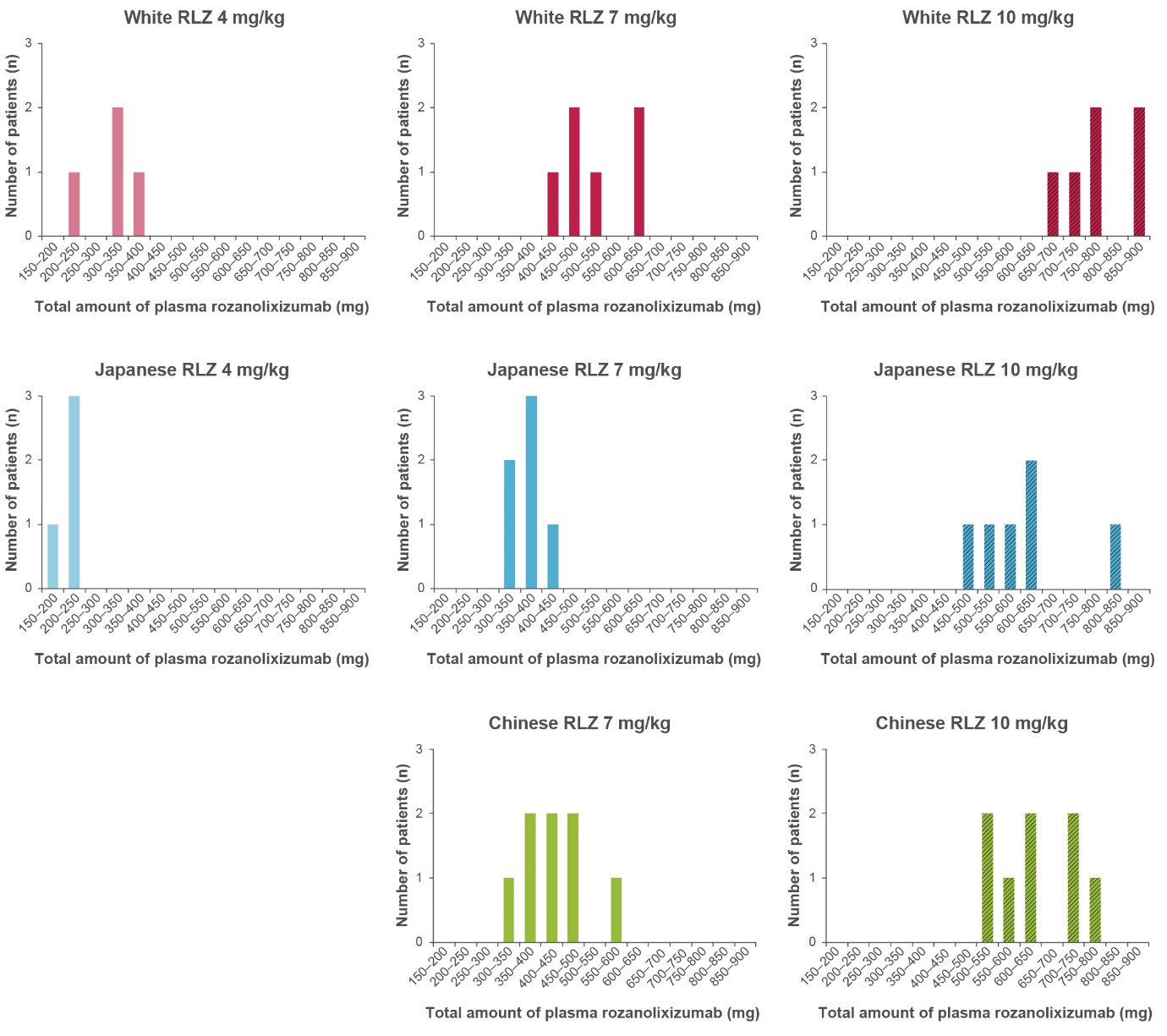


## Supplementary Figure S2: Mean percentage change in IgG1 (A), IgG2 (B), IgG3 (C), IgG4 (D) from baseline in White, Japanese (JPN) and Chinese (CHN) participants, following subcutaneous administration of rozanolixizumab 10 mg/kg


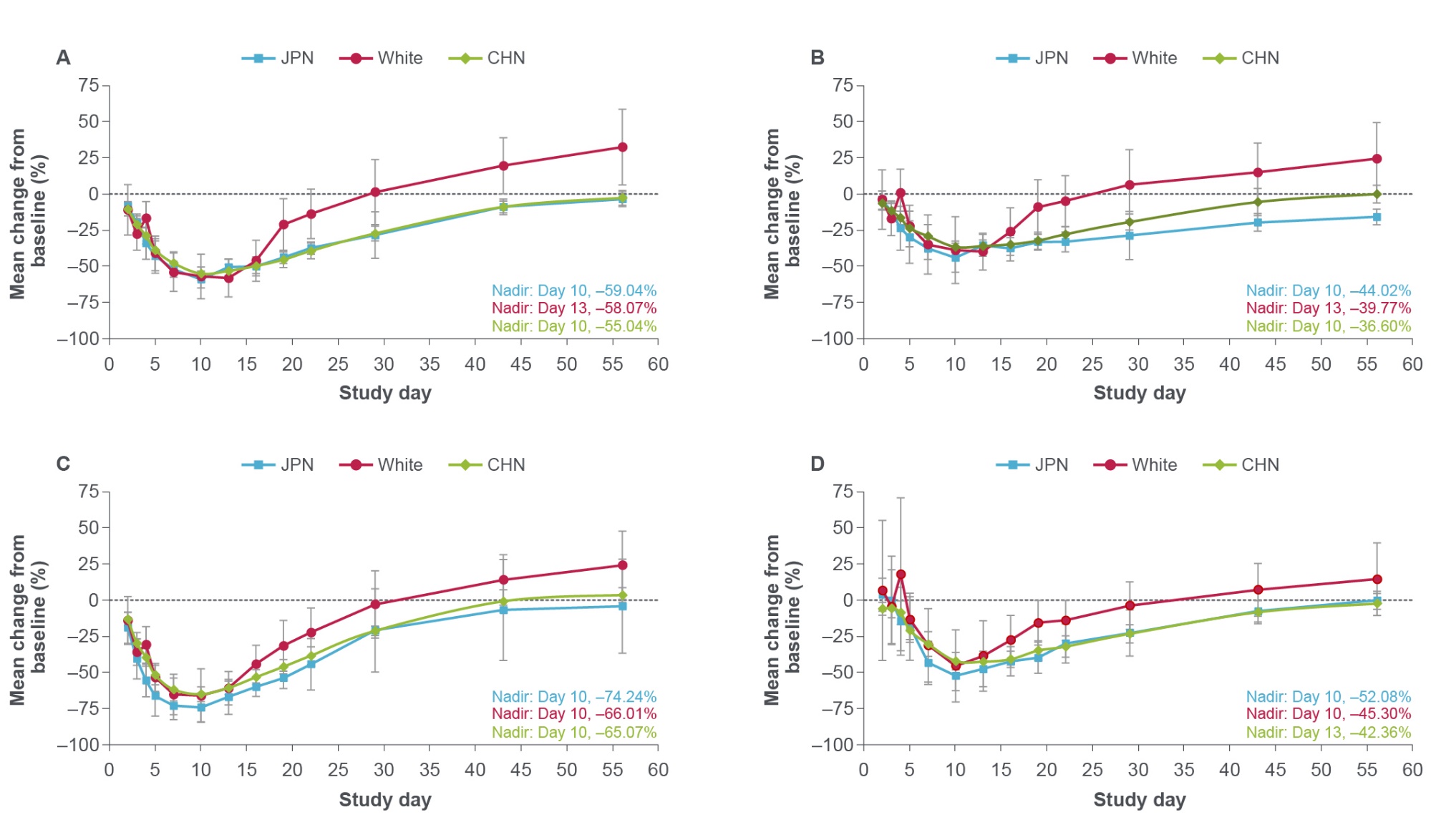


Error bars represent 95% confidence interval.

## Supplementary Figure S3: Mean change in albumin from baseline in White, Japanese (JPN) and Chinese (CHN) participants, following subcutaneous administration of rozanolixizumab 4 mg/kg (A), 7 mg/kg (B) or 10 mg/kg (C), or placebo (D)


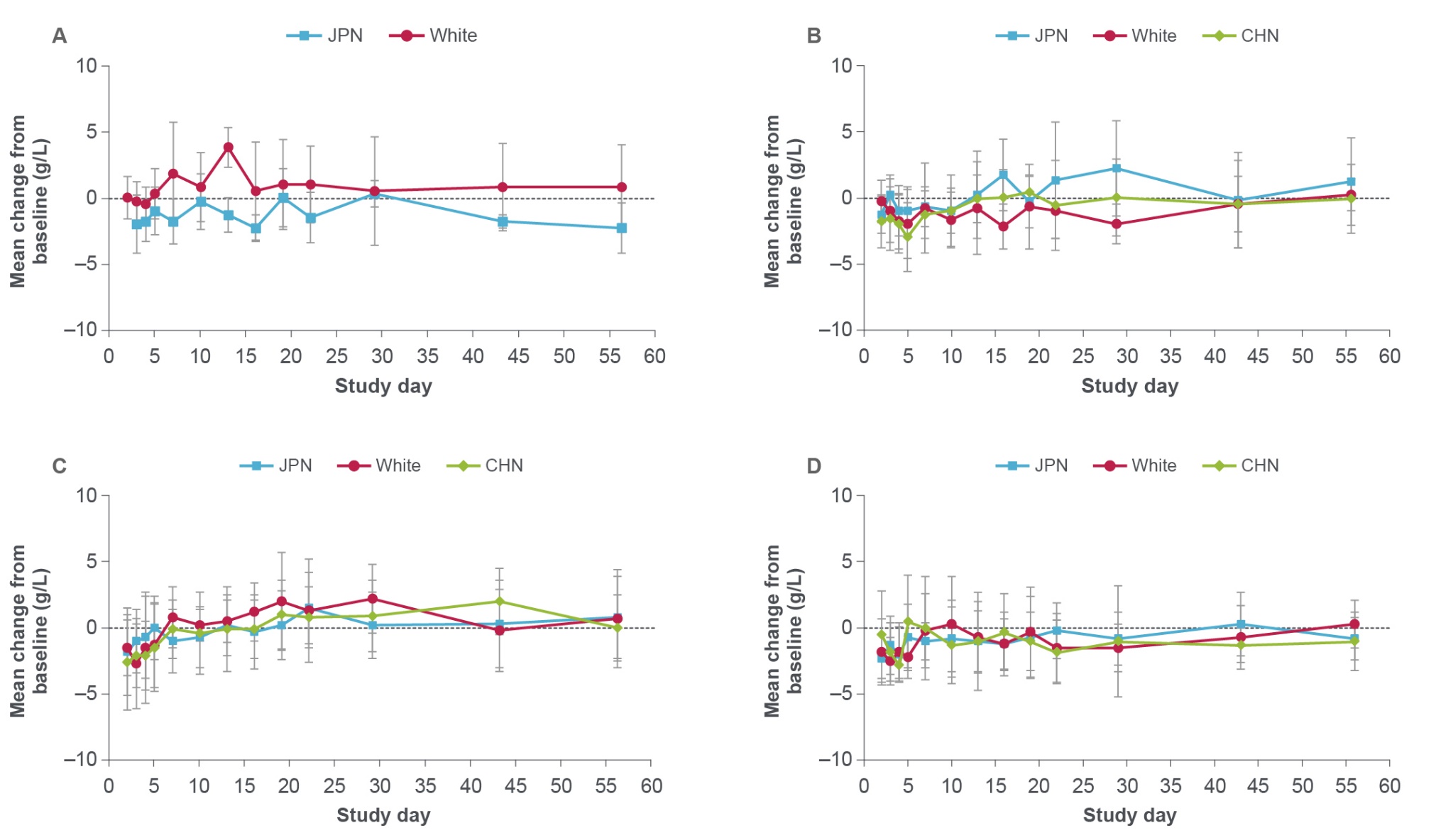


Error bars represent standard deviation.

# Supplementary Methods

***Sample analysis methods for PK data***

For the electrochemiluminescence immunoassay, streptavidin-coated plates were blocked with blocking buffer. Following a wash step, the plate was coated with the capture antibody (biotinylated anti-ID Ab13) and incubated for 1 hour at room temperature (RT, nominally 22°C) with shaking at 600 rpm. After washing, samples, calibrators and controls were added to the wells and incubated for 1 hour at RT with shaking at 600 rpm. The plate was washed, and detection antibody (anti-ID Ab13 Sulfo-tag) was added. After incubation for 1 hour with shaking at 600 rpm, the plate was washed, read buffer was added and the plate analyzed using a MSD® Sector Imager 600. Current passing through the electrodes on the plate initiated the chemiluminescence conferred by the sulfo-tag label and the emitted light was measured by the CCD camera in the instrument. The amount of light generated was directly proportional to the amount of rozanolixizumab in the sample/standard.

The validated range of the assay was 200 to 12,800 ng/mL. The validated curve fit was a four-parameter with 1/Y^2^ weighting. Data were reported in ng/mL to three significant figures. Accuracy (% relative error [RE]) and precision (% coefficient of variation [CV]) were reported to one decimal place.

***Sample analysis methods for PD data***

Samples were collected in 2.5 mL serum separator tubes and were mixed gently by inversion. Samples were then left upright until clotted (approximately 30 minutes) before centrifugation for 10 minutes at 1500 G. The serum was transferred to 5 mL polystyrene specimen tubes and frozen at −80 ⁰C.

Immunoturbidimetric assays were performed using a COBAS c 311 analyzer (Roche) with TRIS buffer (20 mmol/L, pH 8.0; NaCl: 200 mmol/L; polyethylene glycol [PEG]: 3.6 %). Concentration of IgG was determined using an immunoturbidimetric assay (Tina-quant® Immunoglobulin G Gen.2 commercial kit [Roche]). The IgG assay is an immunoturbidimetric procedure that measures increasing sample turbidity caused by the formation of insoluble immune complexes when antibody to IgG is added to the sample.^1^ Sample containing IgG was incubated with a buffer and a sample blank determination performed prior to the addition of IgG antibody. In the presence of an appropriate antibody in excess, IgG concentration is measured as a function of turbidity.

***Sample analysis methods for detection of ADA***

Immunogenicity testing occurred via a tiered analysis approach consisting of a screening, confirmatory, and titration assay.^2^ The false positive rate (FPR) for the screening tier was calculated using baseline samples, after exclusion of samples potentially containing pre-existing antibodies. The screening cut point factor (SCPF) was calculated as 1 .078378 (rounded to 1 .08) on the normalized signal-to-noise (S/N) scale to yield an approximately 5 % FPR. Any sample with a signal >1.08 x mean plate negative control was considered positive in the screening assay and tested in the confirmatory tier of the assay.

For the electrochemiluminescence immunoassay, samples were acid-treated for 120 minutes through a five-fold dilution in acid, after which one volume of acidified sample was further diluted with a four-fold volume of master mix containing biotinylated rozanolixizumab, ruthenium-labeled rozanolixizumab and TRIS buffer pH 9.0 (1.5 M) as neutralization reagent (final minimum required dilution was 20-Fold). Overnight incubation enabled the formation of immunocomplexes, which were immobilized on a blocked MSD streptavidin-coated plate. After a final wash of the plate, read buffer (2X MSD Read Buffer T) was added and the plate analyzed on an MSD Sector S600. An electric current was applied to the bottom of the plate leading to the emission of chemiluminescence (conferred by the ruthenium-labeled rozanolixizumab) that was detected with a CCD camera. The intensity of the chemiluminescence was proportional to the amount of anti-rozanolixizumab antibodies in the sample.

**References**

1. Rifai N, Gubar K, Silverman LM. Immunoturbidimetry: an attractive technique for the determination of urinary albumin and transferrin. *Clin Biochem*. 1987;20(3):179–81.
2. Myler H, Pedras-Vasconcelos J, Phillips K, et al. Anti-drug antibody validation testing and reporting harmonization. *AAPS J*. 2022;24(1):4.
